# Immunization with a recombinant antigen composed of conserved blocks from TSA56 provides broad genotype protection against scrub typhus

**DOI:** 10.1080/22221751.2019.1632676

**Published:** 2019-06-25

**Authors:** Hong-Il Kim, Na-Young Ha, Gwanghun Kim, Chan-Ki Min, Yuri Kim, Nguyen Thi Hai Yen, Myung-Sik Choi, Nam-Hyuk Cho

**Affiliations:** a Department of Microbiology and Immunology, Seoul National University College of Medicine, Seoul, Republic of Korea; b Department of Biomedical Sciences, Seoul National University College of Medicine, Seoul, Republic of Korea; c Institute of Endemic Disease, Seoul National University Medical Research Center and Bundang Hospital, Seoul, Republic of Korea

**Keywords:** Scrub typhus, *Orientia tsutaugmsushi*, vaccine, TSA56, conserved blocks, CD8 T cell

## Abstract

Scrub typhus is an acute febrile disease caused by *Orientia tsutsugamushi* infection. Despite the wide range of approaches explored during the last seventy years, an effective prophylactic vaccine is not yet available. Here, we developed a novel recombinant antigen derived from conserved regions of 56 kDa type-specific antigen (TSA56), a major outer membrane protein responsible for genetic heterogeneity and antigenicity, and evaluated it as a protective vaccine antigen. Our findings demonstrate that immunization with conserved blocks of TSA56 (cTSA56) not only provides protective immunity against lethal challenges with the homologous genotype, but also confers significantly better protection against heterologous genotypes than TSA56. Adoptive transfer of CD4^+^ or CD8^+^ T cells from immunized mice provided significantly enhanced protection against lethal challenge, whereas immune B cells failed to do so, indicating that cellular immunity against the conserved epitopes plays a protective role. Moreover, immunization with a 10-mer peptide mixture, screened from CD8^+^ T cell epitopes within the conserved region of TSA56, provided enhanced protection against lethal challenge with *O. tsutsugamushi*. Therefore, this novel recombinant antigen is a promising candidate for scrub typhus vaccine against a wide range of *O. tsutsugamushi* genotypes.

## Introduction

Scrub typhus is a mite-borne infectious disease caused by *Orientia tsutsugamushi*, an obligate intracellular bacterium. Early clinical manifestations begin with an eschar at the site of mite feeding and regional lymphadenopathy, followed by fever, headache, myalgia, and rash. Delayed diagnosis and treatment with proper antibiotics, primarily due to the nonspecific febrile symptoms, often lead to various systemic illness including multiple organ failure [1]. Disease mortality of untreated patients has been estimated to be approximately 6.0% with a wide range (min–max) of 0–70% [2]. Even though the endemic region of scrub typhus is geographically confined to the Asia-Pacific area [3], there has recently been a new emergence in South America and Africa [4]. It has been estimated that more than a million cases occur annually within the endemic region; the rapid increase of scrub typhus incidence and sporadic outbreaks makes it an emerging public health issue [3].

Despite the wide range of preventative approaches explored during the past 70 years, an effective vaccine for scrub typhus is not yet available. Most vaccine studies, and even natural infection, resulted in short-term protection and immunity to the homologous genotype [5]. Additionally, reinfection with scrub typhus is relatively common in endemic areas, potentially due to the short memory responses and genetic diversity of a major outer membrane protein, 56 kDa type-specific antigen (TSA56) [3,6]. Although the TSA56 protein is an immune-dominant antigen and has long been considered as a vaccine target, its remarkable genetic heterogeneity among *O. tsutsugamushi* genotypes limits cross-protective immunity against heterologous genotypes [5].

Selecting a conserved antigen is one of the primary strategies in generating a clinically effective vaccine providing protective immunity against a wide range of genotypes [5]. Previously, we reported that immunization with ScaA proteins provides protective immunity in mice when challenged with the homologous genotype and significantly enhanced protective immunity against heterologous genotypes [7]. However, this novel antigen also has variability among *O. tsutsugamushi* genotypes and failed to confer wide protective immunity against infection with phylogenetically distant genotypes [7]. Thus, the search for a conserved and effective vaccine antigen continued.

In this study, we developed an artificial recombinant antigen, cTSA56, comprised of conserved blocks (CBs) of TSA56 proteins by amino acid sequence analysis of 206 unique *tsa56* genes [3]. Immunization with cTSA56 itself conferred significant protection against heterologous genotypes, as well as homologous genotypes; its protective efficacy is even better than our previous study using ScaA combined with TSA56 when challenged with heterologous genotype [7]. Adoptive transfer of T cells from cTSA56-immunized mice also provided enhanced protection against lethal challenge, suggesting a protective role of cellular immunity against conserved epitopes within TSA56. Moreover, vaccination with a 39 peptide mixture selected by CD8^+^ T cell epitope screening also conferred significantly better protection against bacterial challenge than non-immune control. These results indicate that cTSA56 is a promising candidate for scrub typhus vaccine against a wide range of *O. tsutsugamushi* genotypes.

## Materials and methods

### Data collection and sequence analysis

206 unique nucleotide sequences encoding the *tsa56* gene were collected from the National Center for Biotechnology Information (NCBI, http://www.ncbi.nlm.nih.gov/) as previously described [3]. Detailed information on the data sets and analytical methods for sequence alignment, phylogenetic relationship, and pairwise identity and similarity matrices of amino acid sequences are available in our previous report [3]. The conserved blocks (CBs) and variable blocks (VBs) of TSA56 sequences, and the degree of consecutive amino acid variation at each position of the 206 aligned sequences were defined by Gblocks software with default settings [8]. Detailed information of the defined blocks is contained in Supplementary Tables S1 and S2. The diversity levels of the defined blocks were assessed by comparison of pairwise similarity calculated by the MatGAT2.1 program [9]. Cophenetic distances, which represents phylogenetic distance between two sequences, were calculated by Ape package [10].

### Preparation of *O. tsutsugamushi*

The Boryong, Karp, and Kato genotypes of *O. tsutsugamushi* were semi-purified as previously described [7]. Briefly, when more than 90% of the cells were infected, as determined by an indirect immunofluorescence antibody technique [7], the cells were collected, homogenized using a glass Dounce homogenizer (Wheaton, Inc., Millville, NJ, USA), and centrifuged at 500 × *g* for 5 min. The supernatant was stored in liquid nitrogen until use.

### Cloning and expression of recombinant antigens

The *tsa56* gene was amplified from the genomic DNA of *O. tsutsugamushi* Boryong genotype by PCR and a recombinant gene encoding cTSA56 (concatenated CBs of TSA56 Boryong) was synthesized after codon optimization (Bionics, Seoul, South Korea). Primers and sequence information of the recombinant proteins are available in Supplementary Table S3. The genes were cloned into pET-28a (Novagen, Gibbstown, NJ, USA) via EcoRI and SalI sites. TSA56 and cTSA56 proteins were purified from *E.coli* BL21 (DE3) harbouring a recombinant plasmid encoding each protein. Following induction with isopropyl β-D-thiogalactoside (0.1 mM, Duchefa, Zwijndrecht, Netherlands) at 16°C for 18 h, the proteins were purified using Ni-nitrilotriacetic acid His-resin (Qiagen, Calrsbad, CA, USA) according to the manufacturer's instructions. After dialysis against phosphate-buffered saline (PBS), purified proteins were treated with endotoxin removal column (Thermo scientific) and endotoxin contamination was determined using the QCL-1000 kit (Lonza, Bloemfontein, South Africa) according to the manufacturer's instructions. All protein contained less than <0.05 EU/mg of endotoxin. The identity and purity (>90%) of proteins were assessed by Western blotting and Coomassie blue staining, respectively.

### cTSA56-derived peptide library

We synthesized 109 overlapping peptides (length: 10 amino acids, offset: two amino acids) spanning the entire cTSA56 sequence (GenicBio Ltd., Shanghai, China). These sequences are summarized in Supplementary Table S4.

### Immunization of mice and *O. tsutsugamushi* challenge

Animal experiments were approved by the Seoul National University Institutional Animal Care and Use Committee (SNU-180727-6-3) and performed in strict accordance with the recommendations in the Guideline for the care and use of laboratory animals. We used 6- to 8-week-old female C57BL/6 mice (Orient Bio Inc., Seongnam, South Korea) for immunization experiments. Mice were subcutaneously immunized three times at two-week interval. 10 μg of purified TSA56 or cTSA56 proteins in PBS absorbed 9:1 ratio with 2% alhydrogel adjuvant (Invitrogen; 90 μl of antigen and 10 μl of 2% alhydrogel) by pipetting for at least 5 min was used for each immunization. For peptide immunization, mice were subcutaneously immunized with 200 μg of peptides mixture emulsified with incomplete Freund's adjuvants (IFA, Sigma-Aldrich). Blood was collected from immunized mice at one week after each immunization to determine serum antibody titre. Two weeks after the final immunization, mice were intraperitoneally (i.p.) or intravenously (i.v.) challenged with the indicated *O. tsutsugamushi* genotype. Body weight and mice survival were monitored for one month after bacterial challenge.

### ELISA

To determine the titre of antibodies specific to TSA56 or cTSA56 in the sera of immunized mice, immunoassay plates (96-well plates; Nunc, Rochester, NY, USA) were coated with 100 μl of purified antigen at a concentration of 1 μg/ml at 4°C overnight. The plates were then blocked for 2 h at room temperature with PBS containing 5% skim milk. 100 μl of serum samples were serially diluted by 2-fold and incubated for 2 h at room temperature. After washing with PBS containing 0.05% Tween20 (PBST), horseradish peroxidase (HRP)-conjugated goat anti-mouse IgG_1_, or IgG_2c_ (Santa Cruz Biotechnology, Santa Cruz, CA, USA) was added and the mixture was incubated for 1 h at room temperature. Wells were washed with PBST and incubated with 3,3′,5,5′-tetramethylbenzidine peroxidase substrate solution (KPL, Gaithersburg, MD, USA) for 7 min. The reactions were stopped by addition of 1M phosphoric acid solution. Absorbance was measured at 450 nm using a microplate reader (Beckman Coulter Inc., Fullerton, CA, USA). Titres were expressed as the inverse of the highest dilution in which an optical density was greater than the mean absorbance plus 3 standard deviations of negative-control sera collected from three naïve mice.

### Adoptive transfer of lymphocytes and sera

The spleens of naïve or immunized mice were used to prepare CD4^+^, CD8^+^ T cells, or B220^+^ B cells. Cells were isolated by the positive magnetic isolation kit (Miltenyi Biotec, Bergisch Gladbach, Germany; CD4^+^ T cells, Cat# 130-049-201; CD8^+^ T cells, Cat# 130-049-401; B220^+^ B cells, Cat# 130-049-501). The purified lymphocytes were pooled and 10^7^ cells of each lymphocyte subset was transferred to naïve recipient mice by i.v. After 24 h, all recipient mice were challenged i.p. with a lethal dose (100 × LD_50_) of the *O. tsutsugamushi* Karp genotype. Positive magnetic selection generated CD4^+^ T cells of more than 90% purity, CD8^+^ T cells of 80% purity, and B cells of 90% purity with less than 2% of other lymphocyte subsets (Supplementary Figure S1). Pooled sera collected from five naïve mice or immunized (TSA56 or cTSA56) mice were injected to naïve recipient mice (150 μl/mouse) by i.p. All recipient mice were challenged i.p. with a lethal dose (100 × LD_50_) of the *O. tsutsugamushi* Karp genotype.

### Flow cytometry

Spelnocytes were stained with antibodies against the indicated surface molecules after blocking on ice for 30 min with ultrablock solution containing 10% rat sera, 10% hamster sera, 10% mouse sera (Sigma, St. Louis, MO, USA), and 10 μg/ml of anti-CD16/32 (2.4G2; BD Pharmingen, Franklin Lakes, NJ, USA). Anti-CD4 (RM4-5) and CD8 (53-6.7) antibodies (eBioscience) conjugated to differential fluorescent dyes were used for flow cytometric analysis. For intracellular detection of IFN-γ and TNF-α, splenocytes (1 × 10^6^ cells) were treated with 10 μg of purified TSA56 antigen for 18 h or 5 μM of peptides for 6 h in humidified CO_2_ atmosphere at 37°C. Cells were treated with Golgiplug (1 μg/ml, BD Bioscience) for the final 5 h. For bacterial stimulation, splenocytes (2 × 10^6^ cells/24-well) isolated from mice were infected with live *O. tsutsugamushi* (4 m.o.i.) for 4 h and further incubated in the presence of tetracycline (0.3 μg/ml) for one day. Then, cells were analysed by flow cytometry after treatment with Golgiplug for the final 5 h. Surface-stained cells were fixed and permeabilized with Fixation and Permeabilization Solution (BD Bioscience), followed by incubation with anti-IFN-γ (XMG1.2; BD Pharmingen) and TNF-α (MF6-XT22, Affymetrix, Cleveland, OH, USA) antibodies conjugated with differential fluorescent dyes. Fluorescence intensities of the stained molecules were examined on a FACS Fortessa II flow cytometer (BD Biosciences). Data were analysed using Flowjo software (Tree Star, Ashland, OR, USA). Gating strategies for the flow cytometric analyses are summarized in Supplementary Figure S2.

### Determination of bacterial load

Bacterial loads of infected tissues were assessed by quantitative real-time PCR (qRT-PCR) as previously described [11]. Briefly, DNA was extracted from the tissue samples using a DNeasy Kit (Qiagen, Gaithersburg, MD, USA), and the bacterial load was determined by using a primer set derived from the 47 kDa gene: p47 forward (5′-AACTGATTTTATTCAAACTAATGCTGCT-3′), p47 reverse (5′-TATGCCTGAGTAAGATACATGAATGGAATT-3′), and detecting probe (5′-6FAM-TGGGTAGCTTTGGTGGACCGATGTTTAATCT-TAMRA). Bacterial loads were normalized to total μg of DNA per ml for the same sample and expressed as the number of 47 kDa gene copies per μg of total DNA.

### Statistical analysis

The data was analysed using the Graph Pad Prism 5.01 software. Statistical analysis was performed using two-tailed Student's *t*-test with 95% confidence interval or one-way analysis of variance (ANOVA) followed by Newman–Keuls *t*-test for comparisons of values among different groups. Data are expressed as the mean ± standard deviation (S.D.). Statistical analysis on survival rates were performed using the Mantel–Cox Log Rank test. A *p*-value of <.05 was considered statistically significant.

## Results

### Characterization of conserved and variable blocks observed in *tsa56* genes

**Figure 1.**
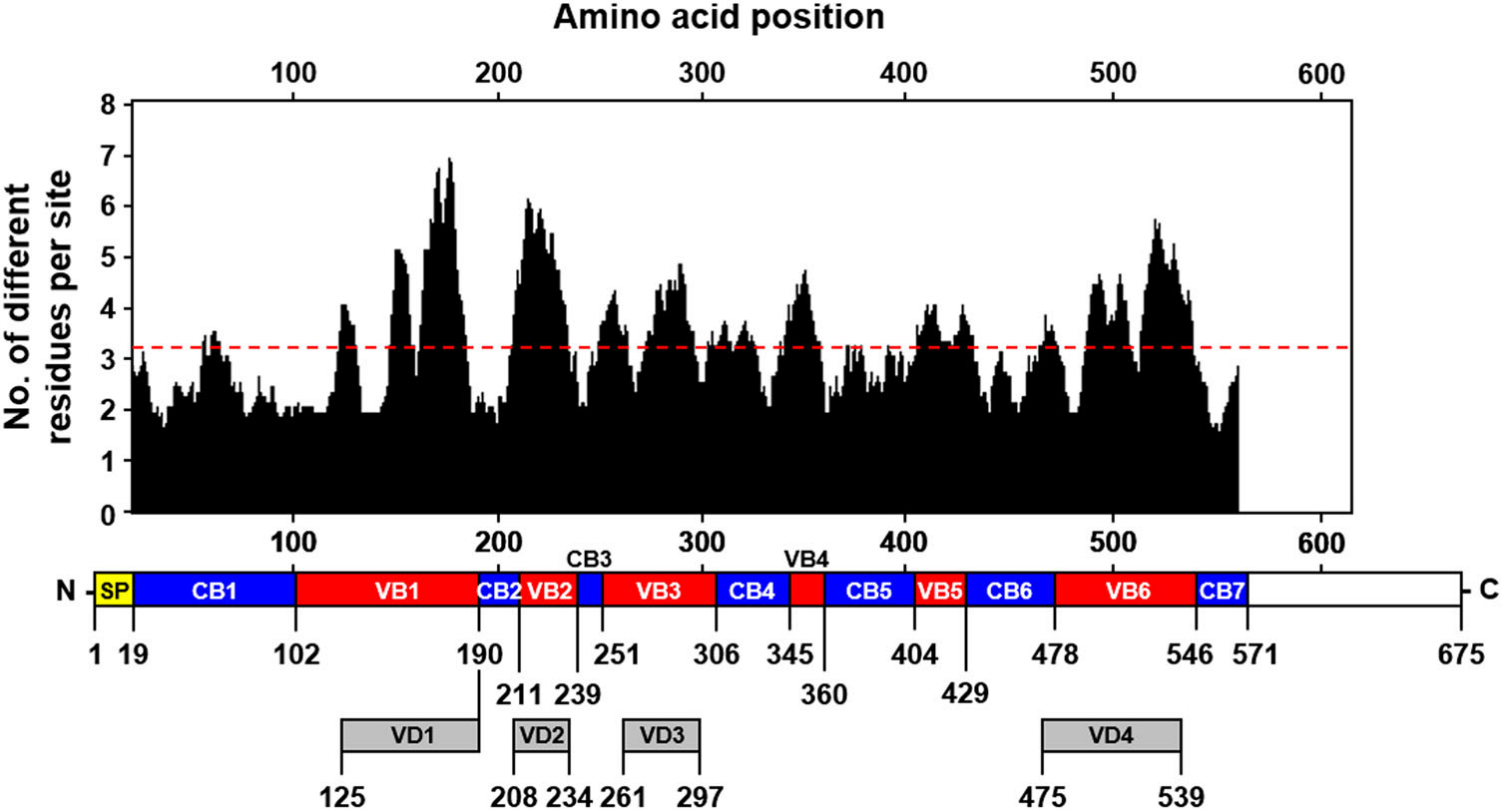
Characterization of conserved and variable blocks in aligned TSA56 sequences. The upper plot shows the moving mean, for a window of 10 amino acid residues, of the number of different amino acids observed at a position in the amino acid alignment using 206 *tsa56* genes (list available at ref. 3). The overall average of sequence variation is noted by the red dashed line. The location of conserved (CB1 ∼ 7, blue) and variable blocks (VB1 ∼ 6, red) annotated in this study and the variable domains (VD1 ∼ 4, grey) defined in a previous work (ref. 12) is shown below the graph. SP, signal peptide.

**Figure 2.**
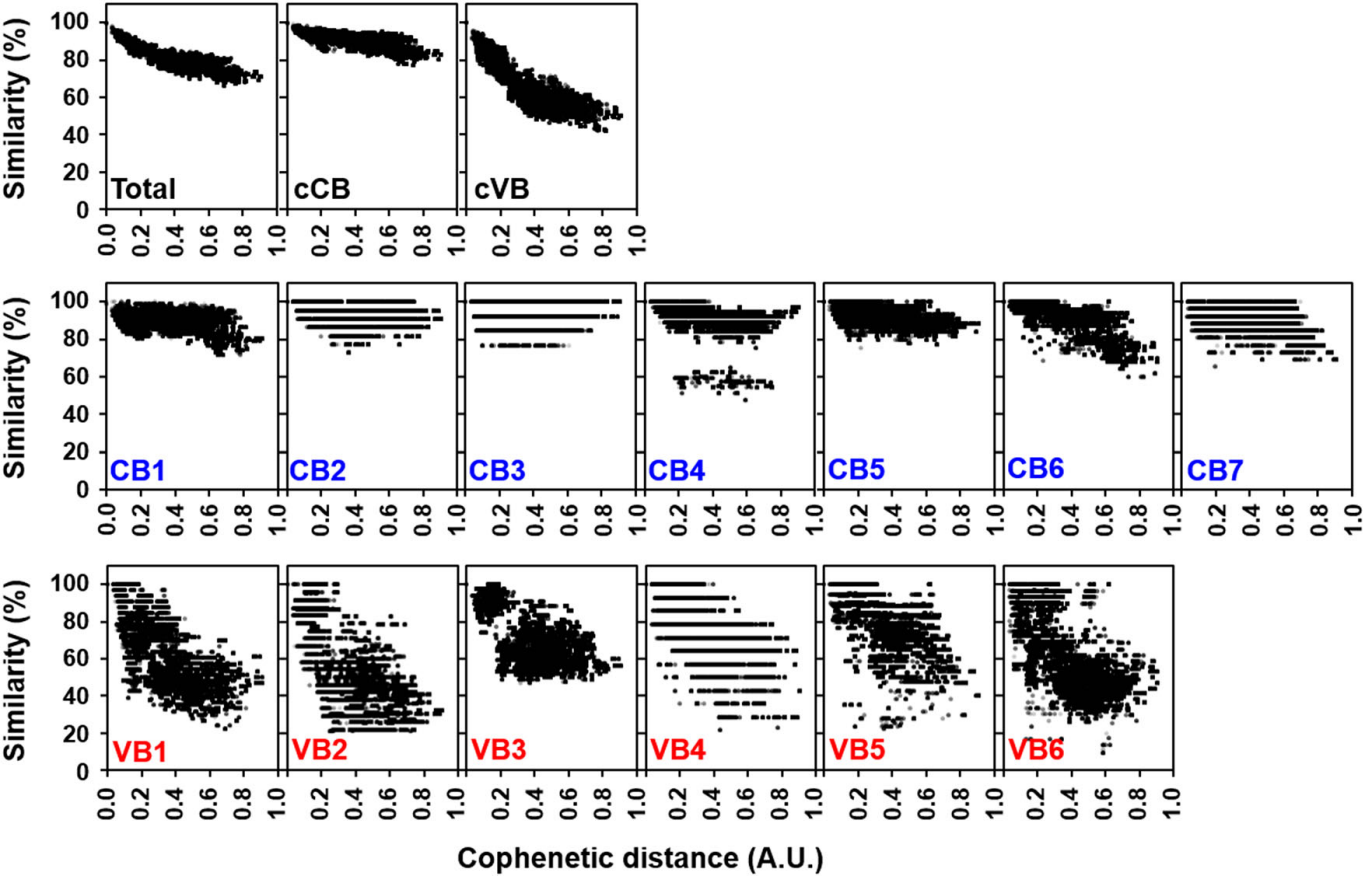
Pairwise comparison of sequence similarity and phylogenetic distance of CBs and VBs in TSA56 sequences. Amino acid similarity of each pair of blocks in TSA56 sequences was measured using MatGAT 2.1. Cophenetic distance, which represents phylogenetic distance between two sequences, was calculated by Ape-package. A pair of sequences, represented by each dot, belonging to different genotypes was chosen randomly for 1000 times and plotted.

To characterize the variation of *tsa56* genes, we defined CBs and VBs based on amino acid sequences by Gblocks software. We also considered variation of amino acids in each position to define the CBs (Supplementary Table S1 and S2). The boundary of each CB was further manually defined to include regions in which amino acids sequences are conserved in more than 90% of the aligned 17 proto-genotypes [3]. Seven CBs, encoding at least 13 consecutive amino acids, were identified and 6 VBs, located between the selected CBs, were defined (Figure 1). When we matched the relative positions of the VBs in the aligned 206 TSA56 sequences, they were found to cover the major variable regions and include four variable domains (VD1∼VD4) defined in a previous study using six genotype sequences [12], and two additional variable blocks (VB4 and VB5). The CBs consist of 17 (CB3) ∼ 84 (CB1) consecutive amino acids with less than four amino acids variation per site. To examine the correlation of sequence variation with phylogenetic distances among the 206 *tsa56* genes, we plotted amino acid similarity with Cophenetic distances [13], calculated from a maximum likelihood phylogeny [3], after pairwise comparison of 206 *tsa56* genes (Figure 2). This revealed that similarity of *tsa56* gene amino acid sequences are negatively correlated with phylogenetic distance, especially in concatenated VBs (cVBs). Minimum pairwise similarity among CBs ranges from 76.9% (CB3) to 47.5% (CB4), whereas VBs showed a greater level of divergence with lower levels of minimum similarity ranging from 46.9% (VB3) to 9.3% (VB6). In addition, there was often a discontinuity in the similarities among the pairs in each CBs and VBs, whereas pairwise comparison of full sequences, as well as cCBs and cVBs, showed continuous distribution in the similarities along with wide range of phylogentic distances. These results strongly suggest that the homoplasy observed among the pairs with a broad range of phylogenetic distances might have resulted from the shuffling of polymorphic segments among diverse genotypes by recombination [3]. The accumulation of subsequent point mutations may have further increased genetic variation while reducing similarities and the degree of discontinuity. Nevertheless, the amino acid sequences of cCBs are more conserved (≥77.6%) than those of cVBs (≥41.8%) among the various genotypes. When we compared the amino acid sequences of 17 proto-genotypes, similarity and identity among the genes of various genotypes range 70.2 ∼ 92.9 and 56.5 ∼ 90.1, respectively, whereas those of cCBs are 80.5 ∼ 96.0 and 67.4 ∼ 94.1, respectively (Supplementary Table S5), indicating that homology of amino acid sequences is generally enhanced in cCBs of TSA56 when compared to full length sequences.

### Immunization with concatenated conserved blocks of TSA56 (cTSA56) provides enhanced protective immunity against various genotype infection

**Figure 3.**
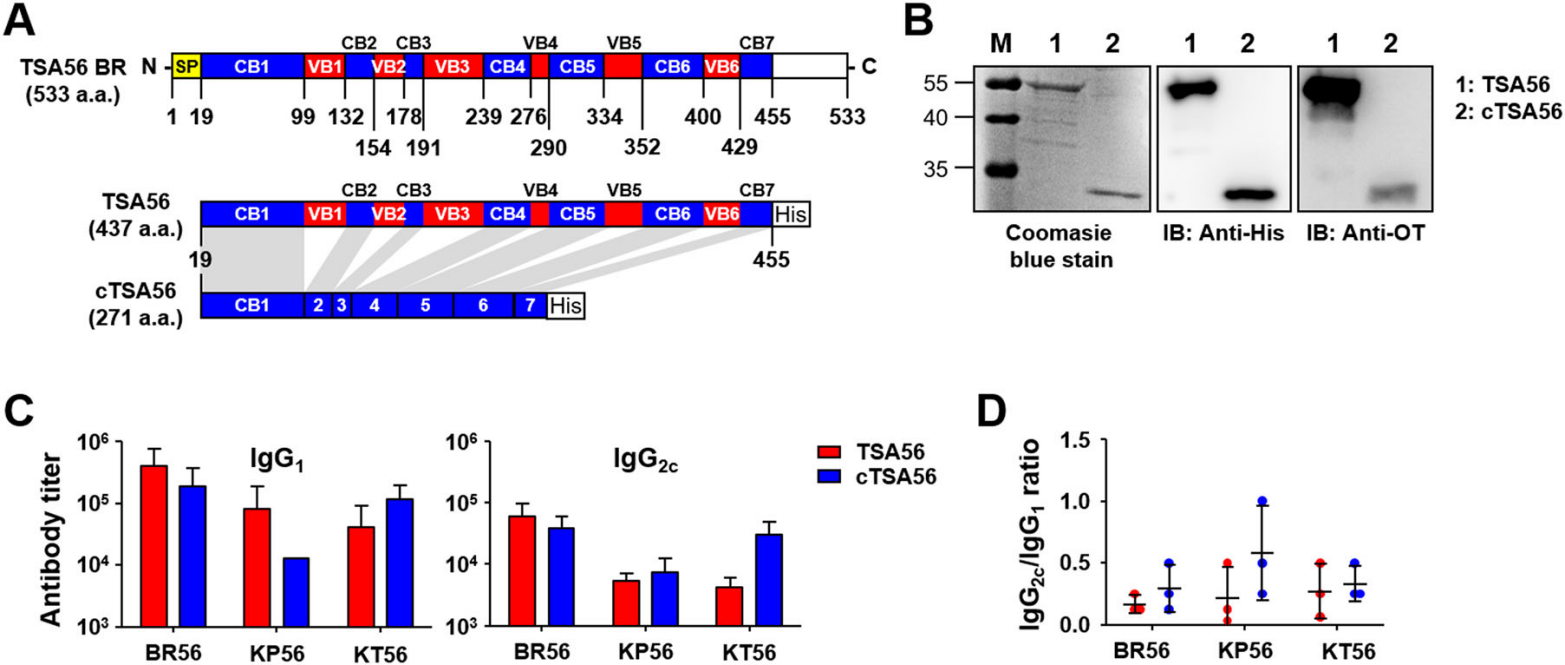
Characterization of purified TSA56 and cTSA56 antigen. (A) Schematic representation of the recombinant TSA56 and cTSA56 used in this study. The amino acid positions of conserved (CB, blue) and variable (VB, red) blocks were indicated. SP: signal peptide. His: 6 × histidine tag. (B) Purified TSA56 and cTSA56 were was resolved by SDS-PAGE, stained with Coomassie blue (left panel), and immunoblotted with anti-His tag antibody (middle panel) or antisera collected from mice recovered from infection with *O. tsutsugamushi* Boryong genotype (right panel). (C) Antibody titres against the indicated antigens were measured by ELISA. Mice (*n* = 3/group) were immunized with TSA56 or cTSA56 three times at two week intervals and sera were collected at one week after the third immunization. (D) IgG2c and IgG1 ratio of the immune sera. Error bar, mean ± S.D.

**Figure 4.**
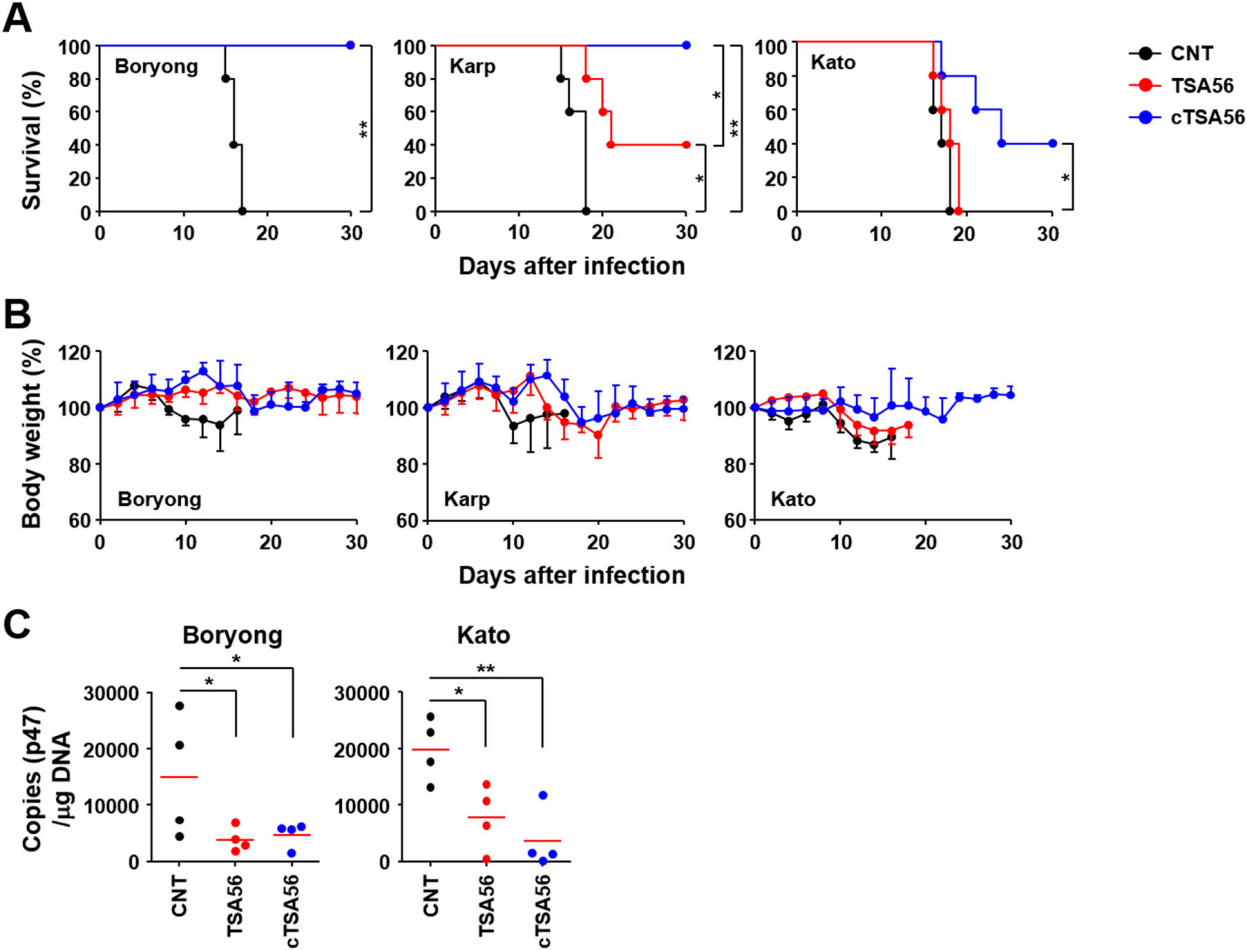
Protective role of cTSA56 against heterologous genotype infection. Mice (*n* = 5/group) were immunized with the indicated antigens and challenged i.p. with 100 × LD_50_ of *O. tsutsugamushi*. Mice were immunized with antigens from the Boryong genotype and challenged with the indicated genotypes. Survival rate (A) and body weight change (B) of mice were observed for 30 d after infection. CNT, mock-immunized; *p < .05; **p < .01. (C) Bacterial loads in the spleens of mice (*n* = 4/group) infected with Boryong or Kato genotype were assessed by qRT-PCR using primer sets detecting the p47 gene of *O. tsutsugamushi*. The infected tissues were collected at 7 days after infection. Black circle, mock-immunized; red circle, TSA56-immunized; blue circle, cTSA56-immunized. Red line, mean; *p < .05; **p < .01.

We cloned *tsa56* gene (Boryong genotype; NCBI accession no.: L04956), including the defined CBs and VBs, and artificially synthesized *ctsa56* gene encoding cCBs (Figure 3(A) and Supplementary Table S3). These cloned genes were expressed in *E. coli* and purified as His-tagged proteins to be tested as vaccine antigens (Figure 3(B)). TSA56 protein reacted well with pooled sera from mice recovered from infection with *O. tsutsugamushi* Boryong genotype, whereas cTSA56 showed reduced reactivity to the same immune sera, potentially due to the loss of immunogenic B cell epitopes [14]. Next, we tested whether immunization with the recombinant cTSA56 could provide protective immunity against *O. tsutsugamushi* infection. We confirmed significant increases of both type 1 (IgG_2c_) and type 2 (IgG_1_) antibodies against TSA56 antigens after three immunizations, although the levels of antibody titres against TSA56 from Boryong, Karp, and Kato genotypes were variable (Figure 3(C,D)). Mice immunized with cTSA56 exhibited a slightly higher IgG2a/IgG1 ratio, but without statistical significance, when compared to mice immunized with TSA56, suggesting that immunization of mice with cTSA56 tends to skew the response towards a Th1 phenotype. Each group of mice were immunized with recombinant TSA56 or cTSA56 antigens derived from the *O. tsutsugamushi* Boryong genotype and then challenged with a lethal dose (100 × LD_50_) of Boryong, Karp, or Kato genotypes (Figure 4). Following infection with *O. tsutsugamushi*, all the mock-immunized mice had succumbed to death by 15 ∼ 18 d after infection regardless of the bacterial genotype. Mice immunized with TSA56 or cTSA56 were protected from the homologous Boryong genotype. Interestingly, survival rate of mice immunized with cTSA56 were significantly better than those of animals vaccinated with TSA56 when challenged with heterologous Karp (100% vs. 40%, *p*-value = .049) or Kato genotypes (40% vs. 0%, *p*-value = .026). These results indicate that immunization with cTSA56 significantly enhanced protective immunity against infection with heterologous genotypes. Even though most of the immunized mice gradually lost weight, especially when challenged with heterologous genotypes, to a similar degree as mock-immunized mice after infection, the time of weight loss slightly delayed and all the surviving immune mice gradually recovered thereafter (Figure 4(B)). In order to investigate inhibitory effect of vaccination on systemic spread of *O. tsutsugamushi* during the acute phase of lethal infection, bacterial loads in spleens of infected mice were assessed at 7 d after infection with homologous Boryong genotype or phylogenetically distant Kato genotype. As shown in Figure 4(C), immunization with TSA56 or cTSA56 significantly reduced bacterial loads in spleens of mice challenged with both genotypes. Even though bacterial loads in mice immunized with both antigens were not significantly different, it is notable that systemic bacterial burden in mice vaccinated with cTSA56 were slightly lower than that of TSA56-immune mice when challenged with Kato genotype.

**Figure 5.**
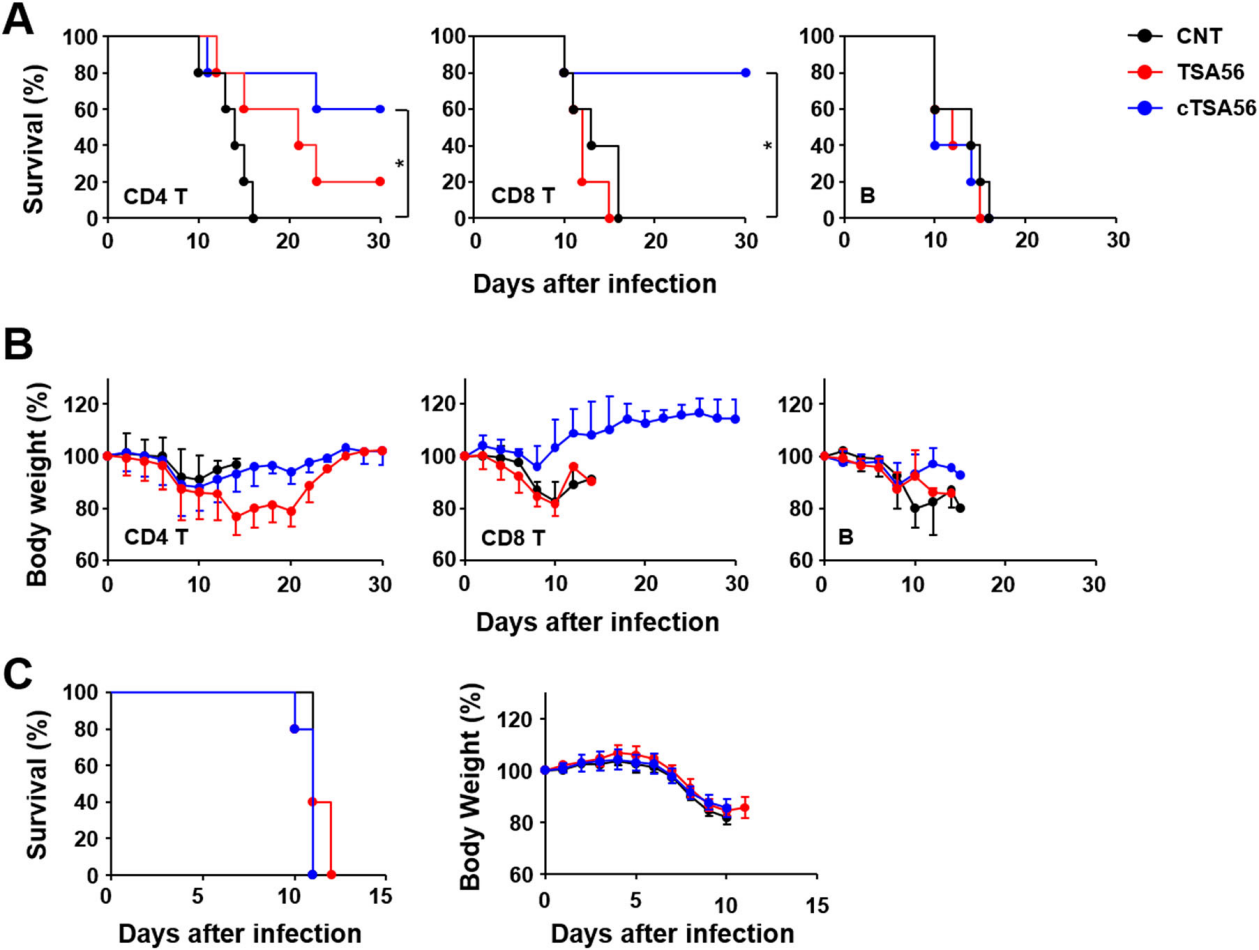
Protective role of immune T cells against heterologous Karp genotype infection. Survival rate (A) and body weight change (B) of mice receiving adoptively transferred indicated populations of splenocytes by i.v. were assessed after challenge with 100 × LD_50_ of *O. tsutsugamushi* Karp genotype. Indicated immune cells purified from splenocytes of mock-immunized (CNT, black circles) or antigen-vaccinated mice (red, TSA56; blue, cTSA56) were transferred to naïve mice (*n* = 5/group) at one day before the lethal challenge. *p < .05; error bar, mean ± S.D. (C) Pooled sera collected from naïve mice (black circle) or mice immunized with TSA56 (red circle) or cTSA56 (blue circle) were injected to naïve recipient mice (150 μl/mouse) by i.p. and then, they were challenged i.p. with a lethal dose (100 × LD_50_) of the *O. tsutsugamushi* Karp genotype. Survival rate (left panel) and body weight change (right panel) of mice receiving pooled sera were assessed.

In order to characterize the immune cell types that confer enhanced protection against the heterologous genotypes, we performed adoptive transfer of lymphocytes after purifying specific subsets of T or B cells from immunized mice. Immune or naive CD4^+^ T cells, CD8^+^ T cells, or B220^+^ B cells purified from splenocytes were adoptively transferred to naive mice. Then, we challenged the recipient mice with a lethal dose (100 × LD_50_) of *O. tsutsugamushi* Karp genotype. All the mice that received naive T cells or B cells succumbed to death between 10 and 16 d after infection (Figure 5(A)). Mice that received immune B220^+^ B cells also succumbed to death after the challenge. However, survival rates of mice that received CD4^+^ T cells (60%) or CD8^+^ T cells (80%) from cTSA56-immunized animals were significantly enhanced compared to those of mice that received naïve lymphocytes. In contrast, all but one of the recipients of immune CD4^+^ T cells and all recipients of CD8^+^ T cells from TSA56-immunized mice died. Trends of body weight changes were consistent with the survival data and the group that received CD8^+^ T cells from cTSA56-immunized mice showed minimum changes (generally less than 10% at the acute phase) in body weight (Figure 5(B)). When mice were received sera from immune mice one day before infection, all the mice group succumbed to death by 10–12 days after infection and there was no difference in body weight change when compared to those of mice received non-immune sera (Figure 5(C)). These results indicate that immune T cells from cTSA56-immunized animals provide enhanced protective immunity against challenge with heterologous genotypes, but immune B cells and sera against TSA56 or cTSA56 failed to do so.

**Figure 6.**
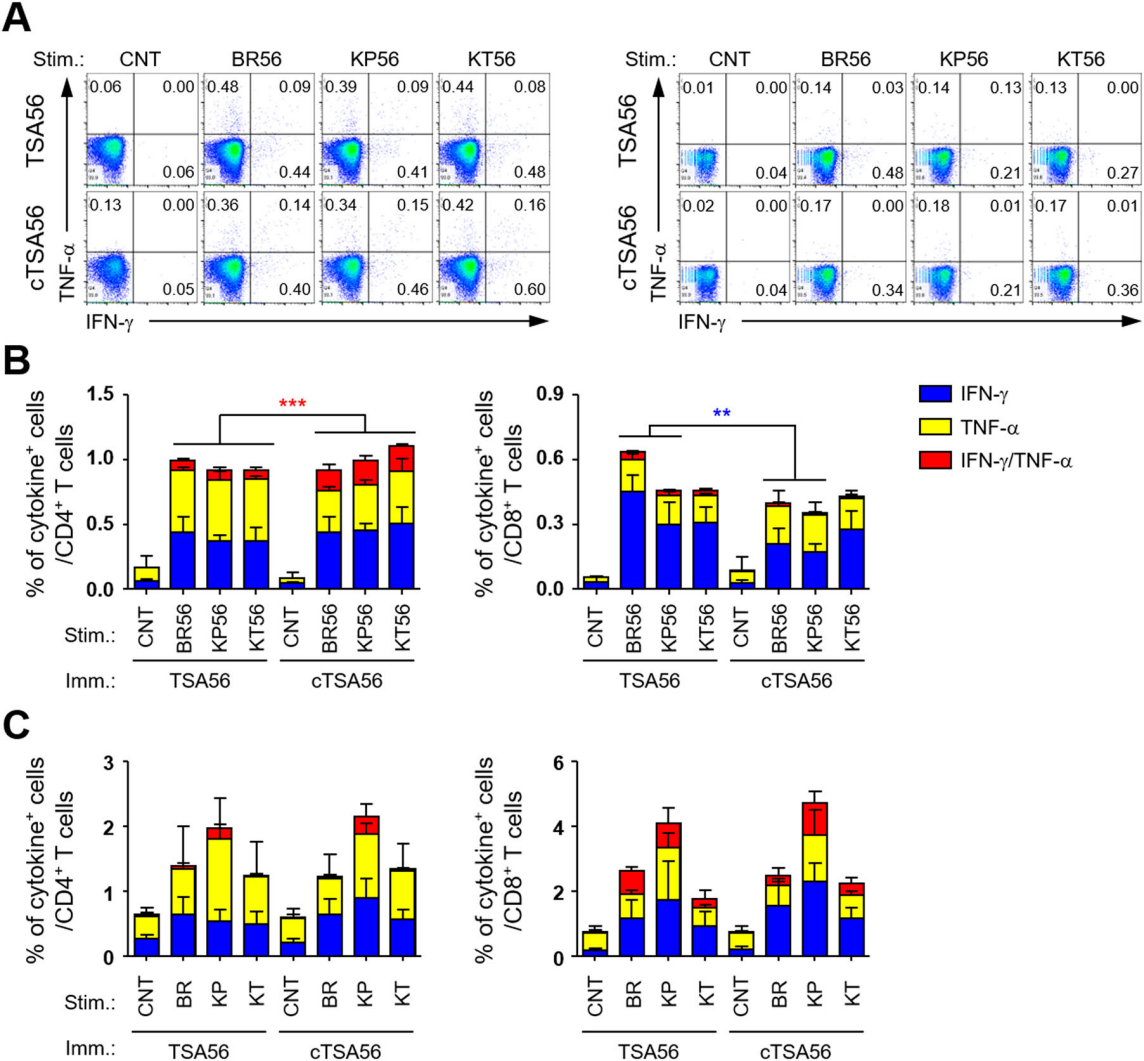
Comparison of antigen-specific T cell responses in mice immunized with TSA56 or cTSA56. (A) Splenocytes were collected from mice at two weeks after the third immunization with the indicated antigen and production of IFN-γ and/or TNF-α by CD4^+^ T (left panels) or CD8^+^ T (right panels) cells were analysed by flow cytometry after stimulation with the indicated antigen (CNT, unstimulated; BR56, TSA56 from Boryong; KP56, TSA56 from Karp; KT56, TSA56 from Kato genotype). Representative flow cytometric results are presented. (B) The percentile of cytokine positive cells among CD4^+^ or CD8^+^ T cell subsets are summarized. Stim., stimulated antigen (the same as in A); Imm., Immunized antigen. Data represent mean + S.D. from duplicate assays with three mice per group. **p < .01; ***p < .001. (C) Splenocytes were collected from mice infected with live *O. tsutsugamushi* (4 m.o.i.) of the indicated genotype (CNT, uninfected; BR, Boryong; KP, Karp; KT, Kato) for 4 h and further incubated in the presence of tetracycline for one day. Then, cells were analysed by flow cytometry. The percentile of cytokine positive cells among CD4^+^ or CD8^+^ T cell subsets are summarized. Stim., stimulated antigen; Imm., Immunized antigen. Data represent mean + S.D. from duplicate assays with three mice per group. Blue box, IFN-γ-positive; yellow box, TNF-α-positve; red box, IFN-γ and TNF-α-positive.

In order to characterize the potential difference in quality of T cell responses in mice immunized with the recombinant antigens, we analysed antigen-specific T cell responses by assessing IFN-γ and TNF-α secreting T cells in an antigen-dependent manner (Figure 6). Splenocytes collected from immunized mice were stimulated with three different TSA56 antigens from the indicated genotypes and the cytokine-positive T cell subsets were analysed by flow cytometry. The frequencies of cytokine-positive T cells in spleens of TSA56 or cTSA56-immunized mice were significantly higher than those of mock-immunized mice, which had similar degree of responses as unstimulated immune splenocytes (data not shown). The levels of CD4^+^ T cells secreting either IFN-γ or TNF-α were not statistically different between TSA56 and cTSA56-immune splenocytes, whereas the frequencies of CD4^+^ T cells positive for both cytokines were significantly increased in mice immunized with cTSA56 when compared with TSA56-immunized mice, regardless of stimulating antigen genotypes (Figure 6(A,B)). Interestingly, IFN-γ-positive CD8^+^ T cells in TSA56-immunized mice were significantly higher than those from cTSA56-immunized mice upon stimulation with soluble TSA56 antigen from Boryong (BR56) or Karp (KP56) genotype, whereas the degree of all other CD8^+^ T cell responses was similar between the two immunized groups. When we assessed T cell responses after infecting splenocytes with three different *O. tsutsugamushi* genotypes *in vitro*, the degree of CD4^+^ or CD8^+^ T cell responses were not significantly different between TSA56 and cTSA56 immunized mice, although the relative frequencies of cytokine-positive T cells were slightly higher in splenocytes infected with Karp genotype (Figure 6(C)). Therefore, T cell responses generated by cTSA56 *in vivo* seem to generally be equivalent to those induced by TSA56 immunization when assessed by immune splenocytes stimulated with soluble protein antigens or infectious *O. tsutsugamushi*. In addition, induction of IFN-γ and TNF-α double positive CD4^+^ T cells was significantly higher in cTSA56 immunized mice than in mice vaccinated with TSA56. However, immunization with cTSA56 induces slightly lower IFN-γ secreting CD8^+^ T cell responses than TSA56 vaccination, suggesting that more immune-dominant CD8^+^ T cell epitopes may be present in variable regions of TSA56.

### Immunization with mixtures of immunogenic peptides derived from cTSA56 provides enhanced protection against *O. tsutsugamushi* infection

**Figure 7.**
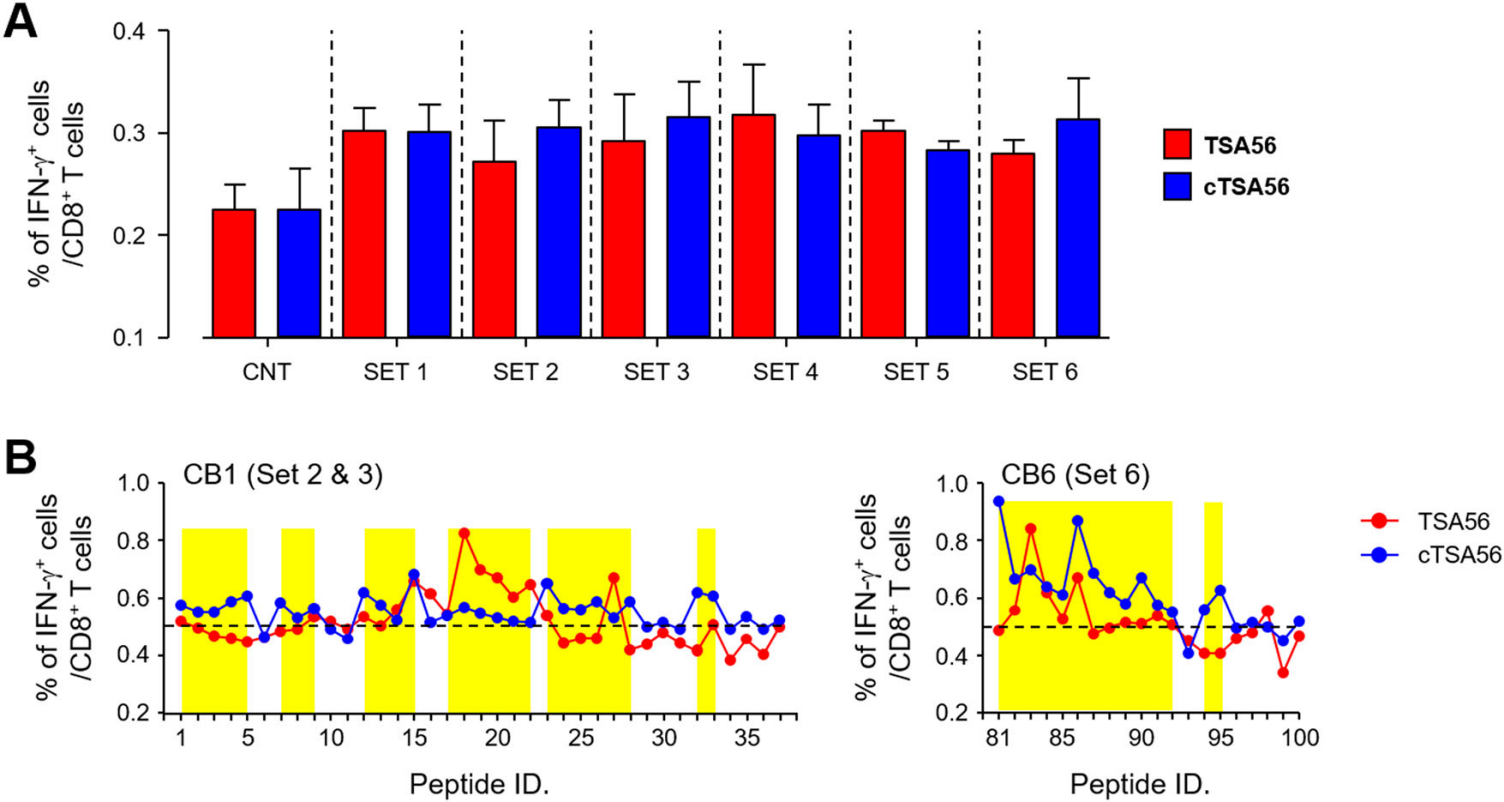
Screening of CD8^+^ T cell epitopes using a 10-mer peptide library derived from CBs of TSA56. (A) Splenocytes were collected from mice at two weeks after the third immunization with the indicated antigen and production of IFN-γ in CD8^+^ T cells were analysed by flow cytometry after stimulation with the indicated sets of peptides. Information of peptide sequences and peptide sets is summarized in Supplementary Table 4. The percentile of cytokine positive cells among CD8^+^ T cells are summarized. Data represent mean + S.D. from duplicate assays with three mice per group. CNT, unstimulated. (B) Immune splenocytes were stimulated with the indicated peptide and production of IFN-γ in CD8^+^ T cells were analysed by flow cytometry. The percentile of cytokine positive cells among CD8^+^ T cells are summarized. Dashed line indicates the baseline frequency (mean + 3 × S.D.) of unstimulated controls in three sets of immune splenocytes. Overlapping peptides showing higher levels of IFN-γ positive CD8^+^ T cells than the baseline frequency are highlighted in yellow.

Since CD8^+^ T cells from Boryong cTSA56 immunized mice provided significantly better protection against heterologous Karp genotype (Figure 5), we next screened CD8^+^ T cell epitopes using a 10-mer peptide library covering the whole cTSA56 sequence (Supplementary Table S4). First, 109 peptides were divided and mixed in six sets, consisting of 15–21 peptides per set, and used to stimulate splenocytes from TSA56 or cTSA56-immunized mice (Figure 7(A)). The frequencies of IFN-γ-secreting CD8^+^ T cells generally increased upon stimulation with the peptide sets when compared to those of unstimulated splenocytes. Even though relative levels of IFN-γ-positive CD8^+^ T cells in peptide-stimulated splenocytes were not significantly different between TSA56 and cTSA56 immune mice, stimulation with peptide sets 2, 3, or 6 induced slightly higher CD8^+^ T cell responses in cTSA56-immunized mice than those from TSA56-vaccinated mice. Therefore, we next measured CD8^+^ T cell responses after stimulation with each peptide included in peptide sets 2 (peptide 1 ∼ 19), 3 (peptide 20 ∼ 37), and 6 (peptide 81 ∼ 100), and identified immunogenic peptides that induce IFN-γ secretion in CD8^+^ T cells from either TSA56 or cTSA56 immune mice, significantly higher than unstimulated controls (mean + 3 × S.D. from three set of immune splenocytes, Figure 7(B)). We identified 39 peptides immunogenic (15 from set 2, 10 from set 3, and 14 from set 6) from this CD8^+^ T cell assay.

**Figure 8.**
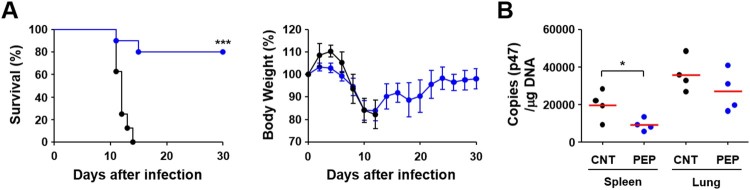
Protection against *O. tsutsugamushi* Karp infection in mice immunized with CD8^+^ T cell epitope peptides. (A) Survival rate (upper) and body weight change (lower) of mice (*n* = 5/group) immunized with 39 peptides selected in Figure 5. Mice were immunized three times at two week intervals and challenged i.v. with 100 × LD_50_ of *O. tsutsugamushi* Karp genotype. ***p < .001. (B) Bacterial loads in the spleens and lungs of infected mice (*n* = 4/group) were assessed by qRT-PCR using primer sets detecting the p47 gene of *O. tsutsugamushi*. The infected tissues were collected at 7 days after infection. Black circle, mock-immunized; blue circle, peptide-immunized. Red line, mean; *p < .05.

Finally, we immunized mice with the selected 39 peptides and challenged them i.p. with lethal doses (100 × LD_50_) of *O. tsutsugamushi* Karp genotype in order to determine whether these CD8^+^ T cell epitopes could provide protective immunity. However, we could not observe any significant protection by immunization with the peptide set and there was no significant difference in survival rate or body weight change (data not shown). Therefore, we adopted milder infection model where mice were infected i.v. with lower but lethal doses (10 × LD_50_ of challenge) of *O. tsutsugamushi* Karp genotype [11,15]. As shown in Figure 8, 80% of mice immunized with the selected peptide set survived, whereas mock-immunized control mice all succumbed to death within 10–13 days after challenge. Even though immunized mice lost weight to a similar degree up to 12 days after infection as mock-immunized mice, all the surviving immune mice gradually recovered thereafter. Bacterial loads were significantly reduced in spleens of immunized mice when compared to those of mock-immunized control at 7 days after infection. Bacterial loads in lungs were also slightly reduced in mice immunized with the peptide set, but without statistical significance. These results suggest that protective CD8^+^ T cell immunity induced by the peptide epitopes derived from CBs of TSA56 might function in the later stage of infection rather than acute phase.

## Discussion

One of the critical hurdles for creating an effective scrub typhus vaccine is the genotypic diversity of *O. tsutsugamushi*, especially in the immune-dominant major outer membrane antigen, TSA56 [1,3]. Major strategies to overcome the diversity barrier include the use of multivalent vaccines containing diverse antigens (i.e. multiple TSA56 antigens from various genotypes, covering the broad range of antigenic heterogeneity) or the application of more conserved antigen and epitopes to target evolutionarily preserved motifs. However, the first strategy may not be appropriate for scrub typhus since *O. tsutsugamushi* genetics feature massive proliferation of mobile genetic elements and repetitive sequences, covering approximately 40% of the whole genome [16], which can drive extensive recombination among various genotypes [3]. Additionally, the mutation rates of major genes encoding outer membrane proteins are generally higher than other house-keeping genes [3]. Moreover, fluctuation and diversification of vector species harbouring *O. tsutsugamushi* in local endemic areas via geographical spread may facilitate genetic recombination among diverse genotypes [3,17]. Therefore, genetic diversification of *O. tsutsugamushi* is continuous, driven by multiple genetic and environmental factors, which is hard to overcome by selecting combinations of multiple variable antigen such as TSA56. In this study, we also confirmed driving forces of genetic heterogeneity of the *tsa56* gene (Figures 1 and 2). Pairwise comparison of CBs and VBs from 206 TSA56 sequences revealed discontinuous homoplasy among pairs with a broad range of phylogenetic distances, suggesting that polymorphic segments are shuffled by recombination (Figure 2), as we and others previously reported [3,17]. In addition, accumulation of point mutations may further contribute to genetic divergence of TSA56, resulting in immense heterogeneity. These unique genetic features might be due to the evolutionary adaptation of *O. tsutsugamushi* to diverse hosts, mite vectors and mammals, during their life cycle. Weak divergence in CBs of TSA56, as well as in core genome including fundamental house-keeping genes, might be due to permanent recombination via lateral gene transfer within mite vectors, whereas VBs of TSA56 might be diverging at higher rates due to selection by the mammalian immune system [3,16–19]. Inversely, less variable CBs of TSA56 might provide key framework of membrane structural proteins, which are highly abundant and may form a cross-linked network across the surface of the outer membrane for bacterial integrity and/or infectivity [12,20–22]. Indeed, a peptide sequence located within CB5 of TSA56, EELRDSFDGYINNAFVNQIHLN, which was identified to bind fibronectin and reduced the bacterial invasion into host cells in our previous study [21], is highly conserved in diverse genotypes (Supplementary Table S2). Therefore, application of the conserved sequence, cTSA56, targeting conserved epitopes derived from less variable structural (and/or functional) frame and thereby providing protective immunity against broad range of genotypes, could be a reasonable strategy as a vaccine antigen. Consistently, we observed immunization with cTSA56 significantly enhanced protection against various genotypes of *O. tsutsugamushi* when compared to TSA56 (Figure 4). Nevertheless, enhanced protection against Karp genotype than Kato by vaccination with cTSA56 derived from Boryong might be due to higher sequence identity between Boryong and phylogenetically close Karp genotype (86.0 ∼ 87.1) than Kato (79.4 ∼ 80.5) (Supplementary Table S5) [3]. Our results also clearly demonstrate that the primary immune components for heterologous protection are the cellular immunity mediated by T cells, rather than B cells and antibodies (Figure 5), as reported by previous studies [1,15,23]. Cellular immunity mediated by cytotoxic CD8^+^ T cells specific to cTSA56 showed the best protection and CD4^+^ T cells partially enhanced protection against lethal challenge with heterologous genotype in our adoptive transfer experiment. Recently, a high proportion of CD4^+^ T cell epitopes with high binding affinity to human leukocyte antigens was predicted to target the conserved region of TSA56 [24]. Our current study also revealed that significantly higher frequencies of CD4^+^ T cells secreting both IFN-γ and TNF-α, representing a poly-functional subset with better quality of T_H_1 responses against intracellular pathogens [25], were induced by cTSA56 immunization than by TSA56 vaccination (Figure 6). In the case of CD8^+^ T cells, however, we observed reduced levels of T cells secreting IFN-γ in cTSA56-immunized mice than in TSA56 immune mice. Though the assessment of CD8^+^ T cell responses by using soluble protein antigen showed relatively higher IFN-γ-positive CD8^+^ T cell responses in mice immunized with TSA56 of Boryong genotype upon stimulation with the same antigen, BR56, when compared to those of other experimental sets, suggesting that dominant CD8^+^ T cell epitopes might be mainly present in VBs, rather than CBs of TSA56 (Figure 6(A,B)). Since we could not also observe significant differences in T cell responses by using an *in vitro* culture system in which two immunization groups were directly infected with *O. tsutsugamushi* genotypes (Figure 6(C)), we further screened CD8^+^ T cell epitopes using a 10-mer peptide library derived from CBs of TSA56 and examined whether the immunogenic epitopes themselves could provide protective immunity against lethal infection (Figure 7). When mice were challenged i.p. with high lethal dose (100 × LD_50_), we failed to observe any significant protection by immunization with the selected peptide epitopes (data not shown). Interestingly, mice immunized with the peptide set showed significantly better survival than mock-immunized mice when challenged i.v. with lower lethal dose (10 × LD_50_), but their initial loss of body weight was similar to that of the control group (Figure 8(A,B)). These results suggest that the initial protective efficacy of antigen-specific CD8^+^ T cells induced by peptide epitopes might be marginal during the acute phase of infection, but the cytotoxic T cell population may efficiently control the systemic spread of *O. tsutsugamushi* at the later stages of systemic infection. Indeed, systemic bacterial loads in spleens of infected mice at 7 days after infection (mice started to die at day 10) were significantly reduced in mice immunized with the peptide epitopes (Figure 8(C)).

Taking all these together, our current data support the following concept of the immunological properties of TSA56 antigen as a vaccine target.

First, CD8^+^ T cell responses specific to the abundant membrane antigen might be the primary effector mechanism for protection against *O. tsutsugamushi* infection. VBs of TSA56 may retain more immunogenic CD8^+^ T cell epitopes, but may continuously mutate due to the selective pressure of adaptive immunity in mammalian hosts. Therefore, these epitopes in VBs might be responsible for homologous protection after natural infection or vaccination. Second, CBs might form the structural and/or functional framework of TSA56 for bacterial integrity and/or infectivity. These sequences may include more immunogenic CD4^+^ T cells epitopes [24], generating polyfunctional CD4^+^ T cells secreting multiple cytokines including IFN-γ and TNF-α, which may confer partial protection against heterologous genotypes sharing the epitopes. However, CD8^+^ T cell epitopes located within CBs may be less immunogenic and/or present in low density. The intrinsic nature of these epitopes may explain the short-lived or poor protection against heterologous genotype infection after natural infection or vaccination with specific TSA56 antigens [1]. Nevertheless, immunization with selected CD8^+^ T cell epitopes could provide enhanced protection against heterologous genotype infections (Figures 5 and 8), suggesting a promising potential as a vaccine target. Presence of potential CD8^+^ T cell epitopes which can stimulate human HLA-A2^+^ T cells in CBs was reported in our previous study [6]. Therefore, induction of CD4^+^ and CD8^+^ T cells specific to the conserved epitopes might be required to enhance vaccine efficacy for scrub typhus. Third, subunit or DNA vaccines, including TSA56, its derivatives, or other antigens such as ScaA and p47, generally failed to inhibit systemic spread of *O. tsutsugamushi* during the acute phase of lethal infection, as measured by weight loss, morbidity, and/or bacteraemia in our and others’ studies, although they generally provided enhanced protection [7,26–28]. In addition, several infection models, including intradermal, intravenous, or intraperitoneal inoculation of the pathogen in mice, partially represented the specific pathology of human scrub typhus, but produced various clinical pathology, disease severity, and susceptibility to the same infectious doses [11,29,30]. A recent report using rhesus macaques as a scrub typhus model also showed a promising potential for evaluation of correlates of protection in both natural and vaccine induced immunity [31]. Application of human HLA-transgenic mice or humanized mice engrafted with functional human immune cells could be additional way to assess efficacy of the antigen-specific T cell responses in the context of human HLA-restriction system [32] Therefore, it would be better to use various mice models simultaneously to assess the vaccine efficacy for scrub typhus and to define the protective immune components before clinical application.

Even though disease morbidity might be associated with route and dose of *O. tsutsugamushi* or their genotype-specific virulence in *in vivo* infection models [11,33], pathogenic properties of the intracellular pathogen, which can replicate within the cytoplasm of phagocytic antigen-presenting cells such as dendritic cells and monocyte/macrophages, may enable evasion of initial antigen-specific T cell responses and cellular autophagy during the acute phase of infection, impairing dendritic cell migration, and reducing expression of MHC class I molecules [34–37]. Moreover, infected phagocytic cells may contribute to bacterial dissemination in the blood stream and lymph nodes as well as further recruitment of monocytes serving as a niche for bacterial replication [1]. In order to overcome these pathologic properties and antigenic diversity of *O. tsutsugamushi*, effective scrub typhus vaccines need to induce potent T cell responses, especially those specific to the conserved epitopes, at the site of initial infection. These can be achieved by selecting a vaccine antigen presenting conserved T cell epitopes, such as cTSA56, use of potent adjuvant favouring cell-mediated immunity, and delivering the vaccine to the natural site of infection to generate intradermal memory T cells [38]. Application of the microneedle system could be a favourable strategy to restrict the initial spread of *Orientia* from the mite bite site [39].

## Supplementary Material

Supplemental Material
