# Supplementary material for: Immunization with a recombinant antigen composed of conserved blocks from TSA56 provides broad genotype protection against scrub typhus
Source: Emerg Microbes Infect. 2019 Jun 25;8(1):946–58. doi: 10.1080/22221751.2019.1632676 (PMC6598529; doi:10.1080/22221751.2019.1632676)
Supplement: Supplemental Material [file TEMI_A_1632676_SM8291.zip › Supplementary Material/temi-2019-0020-20190613173330/doc/Supplementary information_H Kim et al_Final.doc]

***Supplementary information***

**Immunization with a recombinant antigen composed of conserved blocks from TSA56 provides broad genotype protection against scrub typhus**

**Hong-Il Kim^1,2,†^, Na-Young Ha^1,2,†^, Gwanghun Kim^1,2^, Chan-Ki Min^1,2^, Yuri Kim^1,2^, Nguyen Thi Hai Yen^1,2^, Myung-Sik Choi^1^, and Nam-Hyuk Cho^1,2,3,*^**

^1^Department of Microbiology and Immunology, ^2^Department of Biomedical Sciences, Seoul National University College of Medicine, Seoul 03080; ^3^Institute of Endemic Disease, Seoul National University Medical Research Center and Bundang Hospital, Seoul 03080, Republic of Korea

^†^ These authors contributed equally.

* Correspondence: chonh@snu.ac.kr

***Running head:*** A novel vaccine antigen derived from TSA56 for scrub typhus

**Supplementary Figures**

**Supplementary Fig. S1 Purity of T and B cell subsets isolated from mice.** The spleens of naïve or immunized mice were used to prepare CD4^+^, CD8^+^ T cells, or B220^+^ B cells. Cells were isolated by the positive magnetic isolation as described in materials and methods section. The purity of isolated lymphocytes was analyzed by flow cytometry after staining with the indicated surface markers. The positive magnetic selection generated more than 90% purity of CD4^+^ T cells, 80% of CD8^+^ T cells, and 90% of B cells with less than 2% of other lymphocyte subsets

**Supplementary Fig. S2 Gating strategies for T cell analysis by flow cytometry.**
